# Author Correction: SciBet as a portable and fast single cell type identifier

**DOI:** 10.1038/s41467-021-22248-3

**Published:** 2021-03-19

**Authors:** Chenwei Li, Baolin Liu, Boxi Kang, Zedao Liu, Yedan Liu, Changya Chen, Xianwen Ren, Zemin Zhang

**Affiliations:** 1 Peking-Tsinghua Center for Life Sciences, BIOPIC and School of Life Sciences, Peking University, Beijing, China; 2 Analytical Biosciences Limited, Beijing, China; 3 Beijing Advanced Innovation Centre for Genomics, Peking University, Beijing, China; 4 Division of Oncology and Center for Childhood Cancer Research, Children’s Hospital of Philadelphia, Philadelphia, PA 19104 USA

**Keywords:** Machine learning, Transcriptomics

Correction to: *Nature Communications* 10.1038/s41467-020-15523-2, published online 14 April 2020.

The original version of this Article contained errors in Fig. 2g and its associated figure, and the Methods section. In Fig. 2g the analysed data set was identified as GSE86618; it should be GSE81608. In the legend, the reference for this data set was given as reference 26. It should instead be reference 11. As reference 26 is no longer used in this manuscript it has been removed from the reference list. It was 26. Lawlor, N. et al. Single-cell transcriptomes identify human islet cell signatures and reveal cell-type-specific expression changes in type 2 diabetes. *Genome Res.* **27**, 208–222 (2017). In the Methods subsection “Supervised feature selection by E-test” the fourth sentence following equation 3 read “According to Jensen’s Inequality, AM≤GM, and thus the ΔS will always be a non-negative number for each gene.”. It should read “According to Jensen’s Inequality, AM≥GM, and thus the ΔS will always be a non-negative number for each gene”. This has been corrected in both the PDF and HTML versions of the Article.

The original version of this Article also contained errors in the Supplementary Information. In Supplementary Table 3 data set #40 was given as GSE83139^26^; it should be GSE81547^26^. In Supplementary Table 5, the upper data set #8 was given as GSE114725^31^; it should be GSE86146^30^. Also in Supplementary Table 5, the reference given for GSE94820 was given as reference 42; it should be reference 41. Finally, Supplementary reference 29, given as “Zhong, S. et al. A single-cell RNA-seq survey of the developmental landscape of the human prefrontal cortex. 555, (2018)” was incomplete. It should read “Zhong, S. et al. A single-cell RNA-seq survey of the developmental landscape of the human prefrontal cortex. *Nature* **555**, 524–528 (2018)”. The HTML has been updated to include a corrected version of the Supplementary Information.

## Supplementary information

Supplementary information

