# Supplementary material for: SciBet as a portable and fast single cell type identifier
Source: Nat Commun. 2020 Apr 14;11:1818. doi: 10.1038/s41467-020-15523-2 (PMC7156687; doi:10.1038/s41467-020-15523-2)
Supplement: Supplementary file 1 — Supplementary Information [file 41467_2020_15523_MOESM1_ESM.pdf]

SUPPLEMENTARY INFORMATION

SciBet a portable and fast single cell type identifier

Li et al.

Supplementary Figures ---------------- 2-6

Supplementary Tables ---------------- 7-13

Supplementary Notes --------------- 14-18

Supplementary References --------------- 19-22

SUPPLEMENTARY FIGURES

Supplementary Figure 1: Benchmark result of feature selection methods for each dataset ------- 3

Supplementary Figure 2: Benchmark of classifiers for each dataset measured by accuracy score ------- 4

Supplementary Figure 3: Benchmark of classifiers for each dataset measured by balanced accuracy score -------5

Supplementary Figure 4: Benchmark of classifiers for each FPR control pair ------- 6

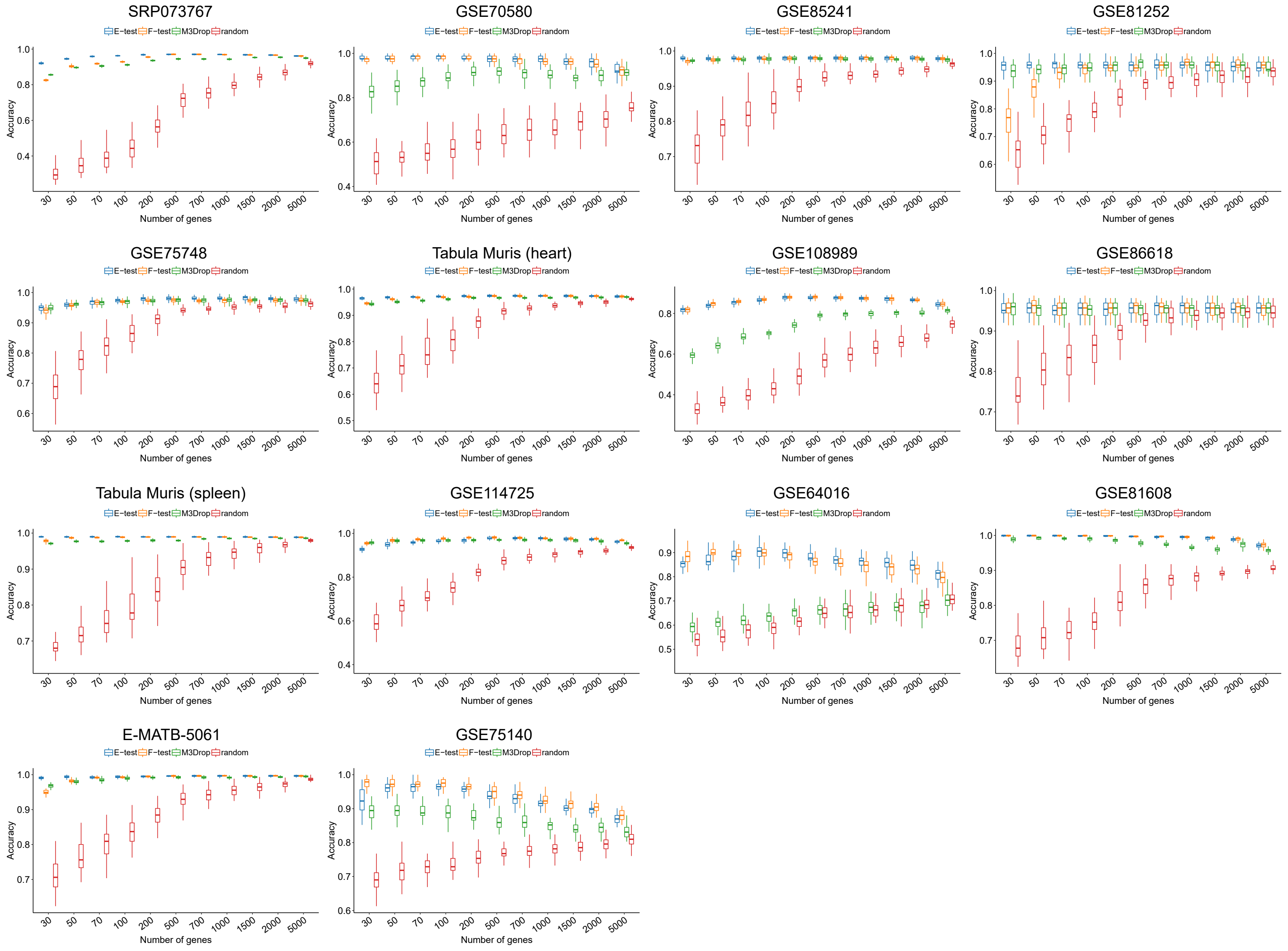

Supplementary Figure 1. Benchmark result of feature selection methods for each of the 14 datasets. For each sub-plot, use scmap on 50 train-test instances (n=50) with different gene numbers, and measure the performance by the accuracy score. Box plot shows the center line for the median, hinges for the interquartile range and whiskers for 1.5 times the interquartile range.

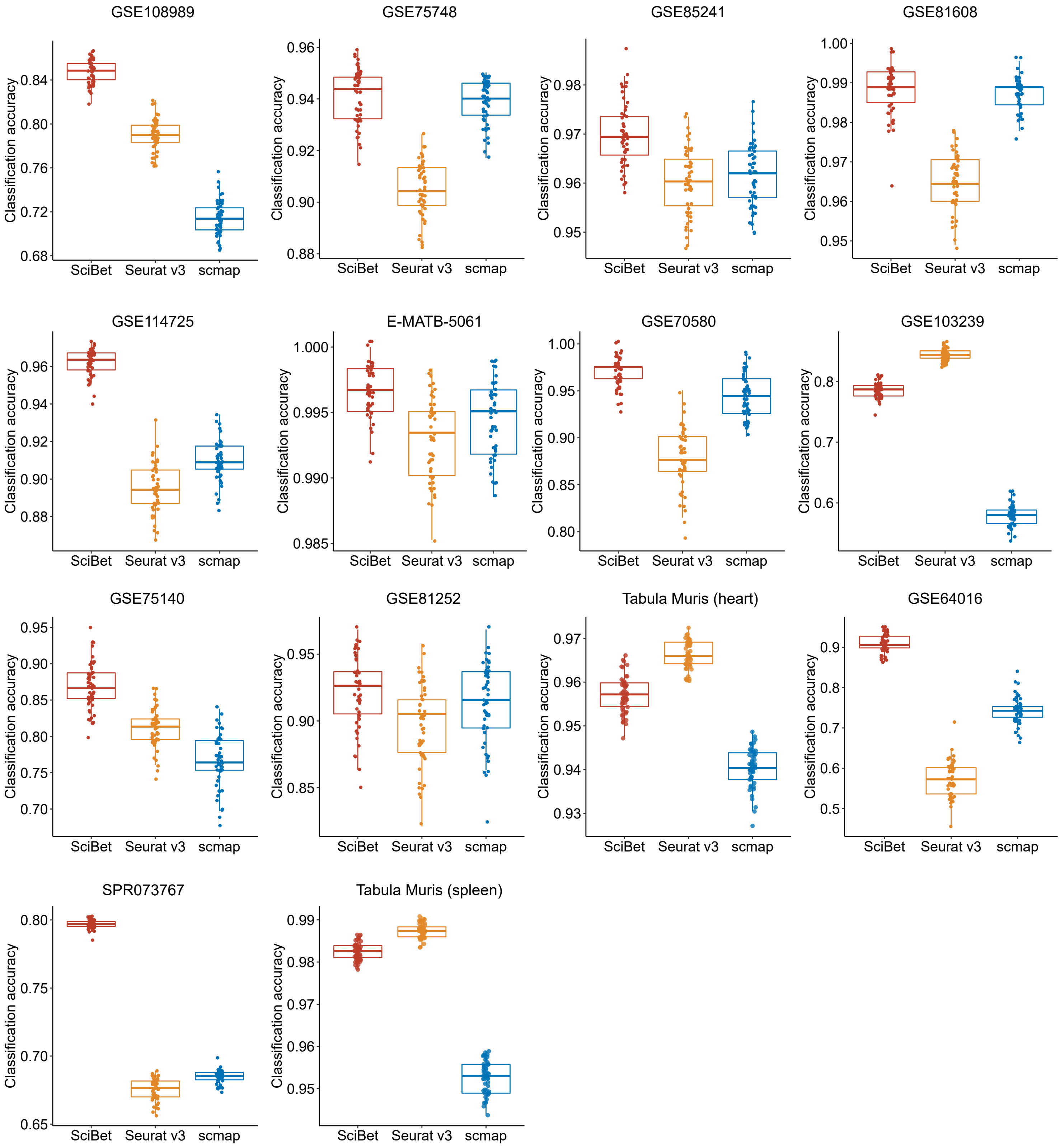

Supplementary Figure 2. Benchmark result of classifiers for each of the 14 datasets. For each sub-plot, benchmark different classifiers on 50 train-test instances (n=50) with 500 genes selected by E-test, and measure the performance by the accuracy score. Box plot shows the center line for the median, hinges for the interquartile range and whiskers for 1.5 times the interquartile range.

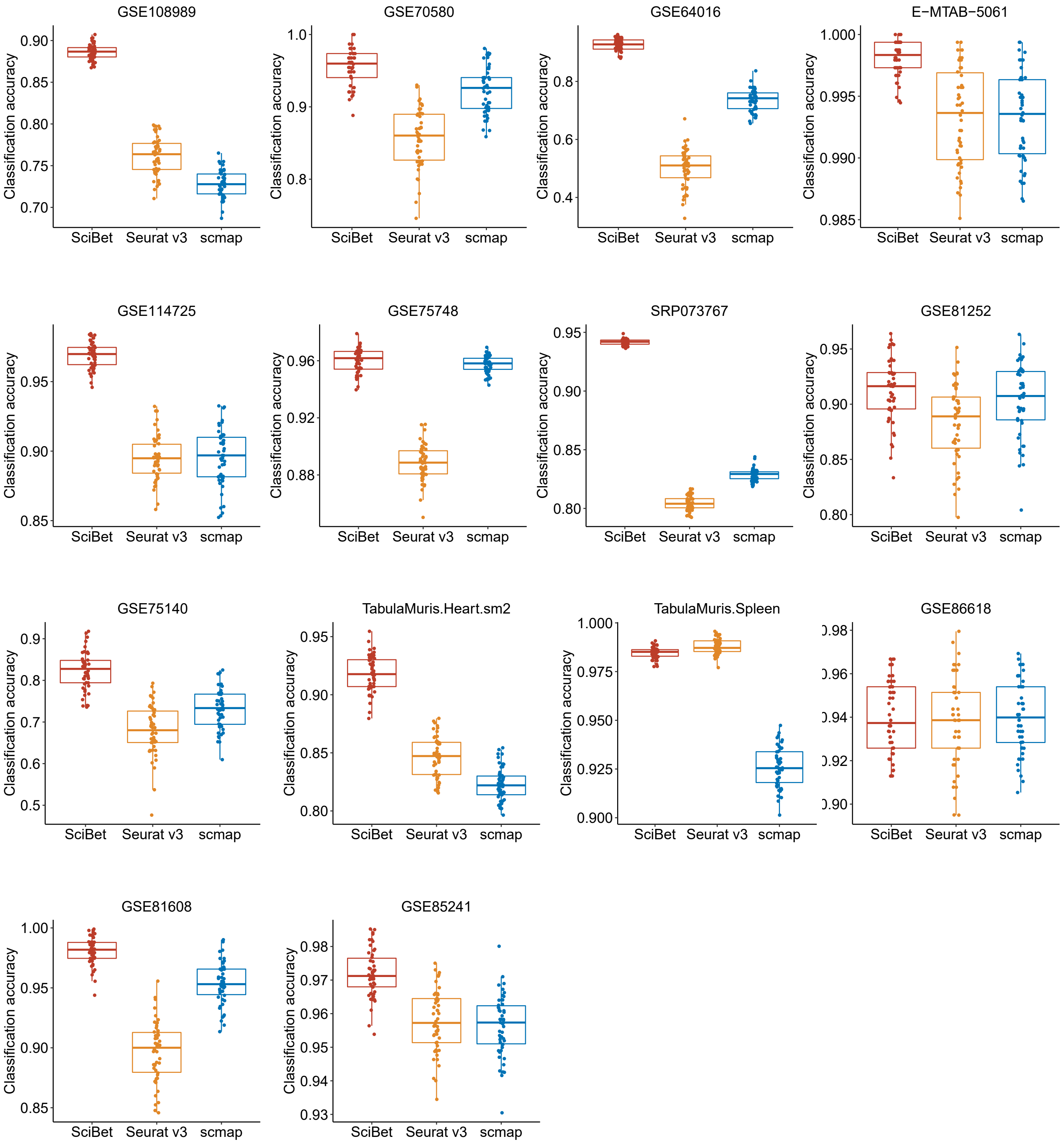

Supplementary Figure 3. Benchmark result of classifiers for each of the 14 datasets measured by balanced accuracy score. For each sub-plot, benchmark different classifiers on 50 train-test instances (n=50) with 500 genes selected by E-test, and measure the performance by the balanced accuracy score. Box plot shows the center line for the median, hinges for the interquartile range and whiskers for 1.5 times the interquartile range.

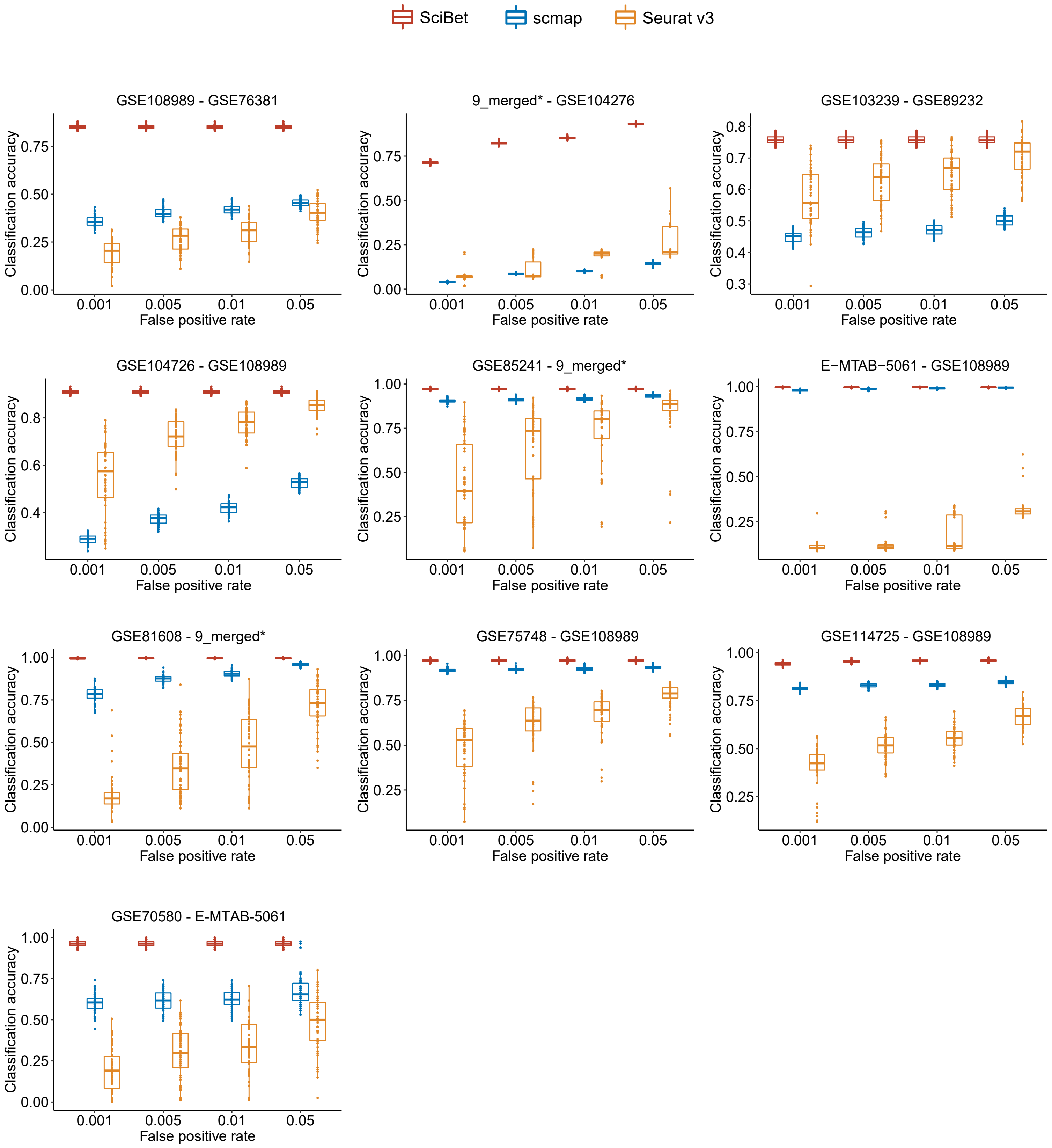

Supplementary Figure 4. Benchmark result of classifiers for each of the 10 FPR control pairs. For each sub-plot, benchmark different classifiers on 50 train-test instances (n=50) with different FPRs, and measure the performance by the accuracy score. Box plot shows the center line for the median, hinges for the interquartile range and whiskers for 1.5 times the interquartile range.

SUPPLEMENTARY TABLES

Supplementary Table 1: Datasets for the cross-validation benchmarks ------- 8

Supplementary Table 2: Datasets for the cross-platform benchmarks ------- 9

Supplementary Table 3: Datasets for building the mock human cell atlas ------- 10

Supplementary Table 4: Datasets for building the null dataset ------- 11

Supplementary Table 5: Datasets for the benchmark of the false-positive control ------- 12

Supplementary Table 6: Datasets of the immune datasets for the E-test case study ------- 13

Supplementary Table 1. Datasets for the cross-validation benchmarks

Num	Dataset	Species	Cell types	Experimental protocol	Number of cells	Number of cell types
1	E-MTAB-5061[39]	human	Human pancreatic cells	Smart-seq2	2038	6
2	GSE64016[6]	human	hESC	SMARTer / Fluidigm C1	460	4
3	GSE114725[31]	human	Immune cells	InDrop	1825	12
4	GSE70580[10]	human	CD127(+) innate lymphoid cells	Smart-seq2	271	4
5	GSE75140[11]	human	fetal neocortex development	SMARTer	472	4
6	GSE85241[2]	human	Human pancreatic cells	CEL-seq2	2018	6
7	GSE75748[13]	human	hESC	SMARTer / Fluidigm C1	1810	12
8	GSE81252[16]	human	Human liver bud	SMARTer	318	5
9	GSE81608[17]	human	Human Islet Cells	SMARTer/C1	1501	4
10	SRP073767[28]	human	Immune cells	10X	17500	7
11	GSE109774[1]	mouse	Spleen	10X	9510	4
12	GSE86618[20]	human	Epithelial cells	SMARTer	540	2
13	GSE109774[1]	mouse	Heart	Smart-seq2	4773	10
14	GSE108989[33]	human	CRC CD8 T cell	Smart-seq2	3099	7

Supplementary Table 2. Datasets for the cross-platform benchmarks

Num	Training set	Experimental protocol	Test set	Experimental protocol
1	GSE84133[3]	inDrop	GSE85241[2]	CEL-Seq2
2	GSE84133[3]	inDrop	E-MTAB-5061[39]	Smart-Seq2
3	GSE84133[3]	inDrop	GSE81608[17]	SMARTer
4	GSE85241[2]	CEL-Seq2	E-MTAB-5061[39]	Smart-Seq2
5	GSE85241[2]	CEL-Seq2	GSE81608[17]	SMARTer
6	E-MTAB-5061[39]	Smart-Seq2	GSE81608[17]	SMARTer

Supplementary Table 3. Datasets for building the mock human cell atlas

Num	Dataset	Tissue	Experimental protocol	Re-map with kallisto	Number of cells
1	GSE85241[2]	pancreatic cells	CEL-Seq2	No	2018
2	GSE84133[3]	pancreatic cells	inDrop	No	8569
3	GSE52529[4]	skeletal muscle myoblasts	SMARTer	Yes	372
4	GSE63473[5]	HEK	drop-seq	No	192
5	GSE64016[6]	hESC	SMARTer / Fluidigm C1	Yes	460
6	GSE65364[7]	human hepatocellular carcinoma	scTrio-seq	Yes	37
7	GSE66053[8]	foreskin fibroblast cells / lung carcinoma	Fluidigm C1	Yes	96
8	GSE67835[9]	brain	Fluidigm C1	Yes	89
9	GSE70580[10]	CD127(+) innate lymphoid cells	Smart-Seq2	Yes	271
10	GSE75140[11]	fetal neocortex development	SMARTer	Yes	472
11	GSE75478[12]	hematopoietic stem and progenitors	Smart-Seq2	Yes	3854
12	GSE75748[13]	hESC	SMARTer / Fluidigm C1	Yes	1810
13	GSE77288[14]	iPSCs	SMARTer/Advantage 2 PCR kit	Yes	2453
14	GSE79920[15]	KD3 myoblasts, myotubes and mononucleated cells	SMARTer/C1	Yes	246
15	GSE81252[16]	human liver bud	SMARTer	Yes	318
16	GSE81608[17]	Human Islet Cells	SMARTer/C1	Yes	1501
17	GSE83139[18]	human pancreatic endocrine cells	SMART-seq	Yes	1189
18	GSE86207[19]	human and chimpanzee neural progenitors	Nextera XT	Yes	52
19	GSE86618[20]	epithelial cells	SMARTer	Yes	540
20	GSE86894[21]	human embryonic stem cells	Smart-Seq2	Yes	3420
21	GSE87237[22]	Human naïve pluripotent stem cells	TruSeq	Yes	288
22	GSE87849[23]	Macrophage	SMARTer	Yes	503
23	GSE89232[24]	dendritic cells, cord blood and blood pre-cDCs	Smart-Seq2	Yes	1543
24	GSE89236[25]	human nasal epithelial cells	5' selective RNAseq	No	96
25	GSE89237[25]	human nasal epithelial cells	5' selective RNAseq	No	96
26	GSE93593[27]	human Interneuron	Smart-Seq2	Yes	1433
27	SRP073767[28]	immune cells	10x genomics	No	17500
28	GSE104276[29]	prefrontal cortex	Smart-Seq2	Yes	2300
29	GSE86146[30]	germ cells	Smart-Seq2	No	2167
30	GSE114725[31]	immune cells	inDrop	No	1825
31	GSE99254[32]	NSCLC T cell	Smart-Seq2	No	12346
32	GSE108989[33]	CRC T cell	Smart-Seq2	No	3099
33	GSE84799[36]	B cell	Smart-Seq2	No	130
34	E-MTAB-5061[39]	pancreatic cells	Smart-Seq2	No	2038
35	GSE77940[37]	B + macrophage + NK + T cell	Smart-Seq2	No	4645
36	GSE103239[40]	fetal digestive tract	STRT-seq	No	5290
37	GSE94820[41]	monocyte	Smart-Seq2	No	2422
38	GSE103154[40]	adult digestive tract	STRT-seq	No	1463
39	GSE98638[43]	HCC T cell	Smart-Seq2	No	5063
40	GSE81547[26]	pancreatic cells	SMARTer	No	635
41	GSE72056[37]	B + macrophage + NK + T cell	Smart-Seq2	No	4645
42	GSE75688[35]	Immune cells	SMARTer	No	515

Supplementary Table 4. Datasets for building the null dataset

Num	Dataset	Species	Tissue	Experimental protocol	Re-map with kallisto
1	GSE85241[2]	human	pancreatic cells	CEL-Seq2	No
2	GSE84133[3]	human	pancreatic cells	inDrop	No
3	GSE52529[4]	human	skeletal muscle myoblasts	SMARTer	Yes
4	GSE63473[5]	human	HEK	drop-seq	No
5	GSE64016[6]	human	hESC	SMARTer / Fluidigm C1	Yes
6	GSE65364[7]	human	human hepatocellular carcinoma	scTrio-seq	Yes
7	GSE66053[8]	human	foreskin fibroblast cells / lung carcinoma	Fluidigm C1	Yes
8	GSE67835[9]	human	brain	Fluidigm C1	Yes
9	GSE70580[10]	human	CD127(+) innate lymphoid cells	Smart-Seq2	Yes
10	GSE75140[11]	human	fetal neocortex development	SMARTer	Yes
11	GSE75478[12]	human	hematopoietic stem and progenitors	Smart-Seq2	Yes
12	GSE75748[13]	human	hESC	SMARTer / Fluidigm C1	Yes
13	GSE77288[14]	human	iPSCs	SMARTer	Yes
14	GSE79920[15]	human	KD3 myoblasts, myotubes and mononucleated cells	SMARTer/C1	Yes
15	GSE81252[16]	human	human liver bud	SMARTer	Yes
16	GSE81608[17]	human	Human Islet Cells	SMARTer/C1	Yes
17	GSE83139[18]	human	human pancreatic endocrine cells	SMART-seq	Yes
18	GSE86207[19]	human	human and chimpanzee neural progenitors	Nextera XT	Yes
19	GSE86618[20]	human	epithelial cells	SMARTer	Yes
20	GSE86894[21]	human	human embryonic stem cells	Smart-Seq2	Yes
21	GSE87237[22]	human	Human naïve pluripotent stem cells	TruSeq	Yes
22	GSE87849[23]	human	Macrophage	SMARTer	Yes
23	GSE89232[24]	human	dendritic cells, cord blood and blood pre-cDCs	Smart-Seq2	Yes
24	GSE89236[25]	human	human nasal epithelial cells	5' selective RNAseq	No
25	GSE89237[25]	human	human nasal epithelial cells	5' selective RNAseq	No
26	GSE93593[27]	human	human Interneuron	Smart-Seq2	Yes
27	SRP073767[28]	human	immune cells	10x genomics	No
28	GSE104276[29]	human	prefrontal cortex	Smart-Seq2	Yes
29	GSE86146[30]	human	germ cells	Smart-Seq2	No
30	GSE114725[31]	human	Breast cancer T cell	Smart-Seq2	No
31	GSE99254[32]	human	NSCLC T cell	Smart-Seq2	No
32	GSE108989[33]	human	CRC T cell	Smart-Seq2	No
33	GSE75688[35]	human	Immune cells	SMARTer	No

Supplementary Table 5. Datasets for the benchmark of the false-positive control

Num	Dataset	Cell types	Negative	Cell types
1	GSE104276[29]	prefrontal cortex	GSE108989[33]	CD8 T cell
2	GSE108989[33]	CD8 T cells	GSE76381[42]	brain
3	E-MTAB-5061[39]	pancreatic cell	GSE108989[33]	CD8 T cell
4	GSE70580[10]	ILC	E-MTAB-5061[39]	pancreatic cells
5	GSE75748[13]	hematopoietic stem and progenitors	GSE108989[33]	CD4 T cell
6	GSE81608[17]	pancreatic cells	9_merged*	immune cells
7	GSE85241[2]	pancreatic cells	9_merged*	immune cells
8	GSE86146[30]	germ cells	GSE108989[33]	CD4 T cell
9	GSE103239[40]	fetal digestive tract	GSE89232[24]	DC
10	9_merged*	immune cells	GSE104276[29]	prefrontal cortex

Num of 9_merged:	Dataset	Cell types
1	GSE99254[32]	T cell
2	GSE108989[33]	T cell
3	GSE84789[36]	B cell
4	GSE77940[37]	B + macrophage + NK + T cell
5	GSE89232[24]	DC
6	GSE94820[41]	DC
7	GSE70580[10]	ILC + NK
8	GSE94820[41]	monocyte
9	GSE87849[23]	macrophage

Supplementary Table 6. Datasets of the immune datasets for the E-test case study

Num	Dataset	Species	Cell types	Experimental protocol
1	GSE99254[32]	human	T cell	Smart-Seq2
2	GSE108989[33]	human	T cell	Smart-Seq2
3	GSE84799[36]	human	B cell	Smart-Seq2
4	GSE77940[37]	human	B + macrophage + NK + T cell	TruSeq
5	GSE89232[24]	human	DC	Smart-Seq2
6	GSE70580[10]	human	ILC + NK	Smart-Seq2
7	GSE87849[23]	human	macrophage	SMARTer

SUPPLEMENTARY NOTES

Note 1: Discussion on the metrics for measuring the classification performance

We collected datasets from the source of the publications with the original cell type annotation. Because the cells were annotated by the publications with the unsupervised workflow, which usually consists of clustering, differential expression and cell type identification based on marker gene of each cluster, cell numbers of different cell types rarely have equal proportions. Such unequal proportions reflect true proportions of cells within each dataset, and thus have different contributions to the global assessment of accuracy. So, we used the *accuracy score*[44] as the default metric, which equals to $\frac{total\ number\ of\ correct\ classification}{number\ of\ all\ cells\ in\ the\ test\ set}$ and also equals to the micro-average recall score of each cell type (i.e., calculating the accuracy of classification for each cell type, and then averaging them with the weight in proportion to the number of each cell type). Here is the derivation:

If we have a dataset with n cell types and for each cell type i, we have assigned a_i and b_i cells with the correct and wrong cell type in the test set, respectively: *accuracy score* $=\frac{total\ number\ of\ correct\ classification}{number\ of\ all\ cells} = \frac{\sum a_i}{\sum a_i + \sum b_i}$. We can also calculate the recall score for each cell type: $recall_i = \frac{number\ of\ correct\ classification\ for\ cell\ type\ i}{total\ number\ of\ cell\ type\ i\ in\ the\ test\ set} = \frac{a_i}{a_i+b_i}$, and $weight_i = \frac{number\ of\ cell\ type\ i}{number\ of\ all\ cells} = \frac{a_i+b_i}{\sum a_i + \sum b_i}$. So, the micro-average recall $=\sum(acc_i * weight_i) = \sum(\frac{a_i}{a_i+b_i} * \frac{a_i+b_i}{\sum a_i+\sum b_i}) = \frac{\sum a_i}{\sum a_i+\sum b_i}$= accuracy score.

We also applied the balanced accuracy score[44] (the macro-average recall score) to measure the overall performance, which equals to $\frac{1}{n}\sum \mathrm{recall}_i$, to account for the rare cell types, where each cell type in the test set has equal contribution.

Note 2: Detailed derivation and discussion for E-test

Note 2.1: Calculation of entropy in a given cell type

We applied the strategy proposed by Splatter[45] and Saver[46] to model the observed expression Z_{ic} for gene i (ranges from 1 to m) in cell c (ranges from 1 to C) with the identical cell type as following：

$$Z_{ic} \sim \text{Possion}\ (\lambda_i * s_c),\ \lambda_i \sim \text{Gamma}\ (\alpha_i,\ \beta_i)$$

, where λ_i represents the Gamma-distributed true expression of gene i in cell c, and s_c represents the size-factor (can be calculated by $\sum_c Z_{ic}$) of cell c accounting for the sequencing depth. Then we approximately estimate the Poisson rate $\lambda_i * s_c$ directly by Z_{ic} using the moment estimation. Thus, for each cell we will obtain $\lambda_{ic}=\frac{Z_{ic}}{s_c}$ as C observations for λ_i, which is in accordance with the size-factor normalized expression described in part <u>Data collection and processing</u> of section <u>Methods.</u>

We applied the differential entropy from information theory to measure the dispersion degree of the distribution. The Shannon entropy S_i of the Gamma-distributed true expression λ_i can be calculated by the equation[47]:

$$S_i = \alpha_i - \ln \beta_i + \ln\ \Gamma\ (\alpha_i) + (1-\alpha_i)\psi\ \ (\alpha_i) \quad \text{----------------(1)}$$

, where ψ represent the digamma function. Then we replace β_i with its maximum likelihood estimation as $\hat{\beta}_i = \frac{\alpha_i}{E(\lambda_i)}$, where $E(\lambda_i)= \frac{1}{C}\sum_c \lambda_{ic}$ served as the unbiased estimation of λ_i. Thus, formula (I) can be further derived into:

$$S_i = \alpha_i - \ln \alpha_i + \ln\ \ E(\lambda_i) - \ln\ \Gamma\ (\alpha_i) + (1-\alpha_i)\psi\ \ (\alpha_i) \quad \text{-----------(2)}$$

We denote $h_i = \alpha_i - \ln\alpha_i + \ln\Gamma(\alpha_i) + (1-\alpha_i)\psi(\alpha_i)$ ------------(3)

as the function of the gene-specific parameter α_i, and denote the mean normalized expression over C cells as $X_i = E(\lambda_i) = \frac{1}{C}\sum_c \lambda_{ic}$. Thus, Supplementary Equation (2) can be derived into

$$S_i = \ln E(\lambda_i) + h_i = \ln X_i + h_i \quad \text{------------(4)}$$

Note 2.2: Calculation of entropy of different cell types

In Supplementary Note 2.1, we obtained the Supplementary Equation (4) for the calculation of entropy of gene i in a given cell type. For cell *c* (ranges from 1 to C_j) belonging to cell type j, we define $X_{ij} = \frac{1}{C_j}\sum_{c\in j}\lambda_{ic}$ as the mean normalized gene expression over cells belonging to cell type j, and calculate the entropy S_{ij} of gene i in cell type j as:

$$S_{ij} = \ln X_{ij} + h_{ij} \quad \text{------------ (5)}$$

, where $h_{ij} = \alpha_{ij} - \ln\alpha_{ij} + \ln\Gamma(\alpha_{ij}) + (1-\alpha_{ij})\psi(\alpha_{ij})$ ------------(6)

We then assume that $h_{ij} = h_i$, that is, h is gene-specific but not cell type-specific. Then we obtained the Equation (1) in Main Text: $S_{ij} = \ln X_{ij} + h_i$. The assumption of $h_{ij} = h_i$ is equivalent to another assumption proposed by Lun et al.[48], where they modeled the differential expression among multiple groups with fixed fold changes. Here is the derivation: We denote $\lambda_{i,c\in j}$ as the observations of the Gamma-distributed true expression λ_{ij}~Gamma $(\alpha_{ij}, \beta_{ij})$ of gene i in cell type j. For gene i, if we multiply λ_{ij} with a fixed constant $F_{i,j\to j'}$ as the fold change of gene i, from cell type j to j′, as $\lambda_{ij'} = F_{i,j\to j'} * \lambda_{ij}$. Thus, $\lambda_{ij'}$ will follow Gamma $(\alpha_{ij}, \beta_{ij}/F_{i,j\to j'})$, according to the scaling property of the Gamma distribution (See the following lemma in Note 2.3). Here we note that the Gamma-distributed $\lambda_{ij'}$ and λ_{ij} share the identical α_{ij}. Thus, for all cell types, the shape parameter α_{ij} is not cell type-specific, as $\alpha_{ij} = \alpha_i$. According

to Supplementary Equation (6), h will also be the gene-specific but not cell type-specific variable in accordance with α.

Note 2.3: The scaling property of the Gamma distribution

Definition: if X ~ Gamma (α, β), then Y = kX ~ Gamma (α, β/k) for any k>0.

Proof: The probability density function for X is defined as:

$$f_X(x) = \frac{\beta^\alpha}{\Gamma(\alpha)} x^{\alpha-1} e^{-\beta x}, \quad \text{for all x>0}$$

We then let k as a positive constant, and thus the Jacobian of the transformation will be $\frac{dX}{dY} = \frac{1}{k}$.

So, $f_Y(y) = \frac{dX}{dY} * f_X\left(\frac{y}{k}\right) = \frac{1}{k} * \frac{\beta^\alpha}{\Gamma(\alpha)} * (\frac{y}{k})^{\alpha-1} * e^{-\frac{\beta y}{k}} = \frac{(\frac{\beta}{k})^\alpha}{\Gamma(\alpha)} * y^{\alpha-1} e^{-\frac{\beta}{k} * y}$, for all y>0.

Now $f_Y(y)$ is exactly the probability density function of Gamma (α, β/k).

Note 2.4: Permutation test for obtaining the significance of ΔS

ΔS_i, the total entropy difference of gene i is defined as Equation (3) in the main text, as $\Delta S_i = \sum_{j=1}^{n}(S_{i0} - S_{ij}) = \sum_{j=1}^{n}(lnX_{i0} + h_i - lnX_{ij} - h_i)$, where the significance of ΔS can be approximated by the permutation test (randomly permutate the cell group labels, calculate ΔS for each permutation and find the percentile of the actual ΔS), which may take long time. Here we show a parametric method to accelerate this process by replacing the permutating step, as following:

Under the null hypothesis that all cells from the pre-defined groups are randomly sampled from the the same cell population, each cell type j will have the identical mean size-factor normalized expression X_{ij}, which follows a normal distribution N(μ_i, σ_i) according to the central limit theorem. Here we can denote the unbiased estimation for the parameters as $\hat{\mu}_i = \frac{1}{n} * \sum_{j=1}^{n} X_{ij}$ and $\hat{\sigma}_i = \frac{1}{n-1} * \sum_{j=1}^{n}(X_{ij} - \hat{\mu}_i)^2$. Notably, $\hat{\mu}_i$ is exact the X_{i0} , the mean size-factor normalized expression of the null group 0, as we defined in the main text. For each round

of permutation, we can generate n X_i observations from N(μ_i, σ_i) with the constraint that $\hat{\mu}_\mathrm{i} = \frac{1}{n} * \sum_{j=1}^{n} X_{ij}$ (this process equals to generating n-1 X_i observations and calculate another by the n-1 observations and $\hat{\mu}_\mathrm{i}$). Then, we can calculate ΔS according to formula (***).

Note 3: Discussion on whether to use the cell type prior probability in SciBet model training

The proportion of each type of cell in the data measured by a single cell sequencing does not necessarily and correctly reflect the prior probability of appearance. For example, if a piece of tissue is sequenced without any sorting, then the proportion of each cell type in the results of single-cell sequencing can reflect the prior probability. However, if certain artificial filtering (such as Fluorescence-activated cell sorting to select cells highly expressing certain surface protein) is performed, or the dataset is integrated from different batches or studies, then the final cell type ratio at this time cannot correctly reflect the prior probability of the appearance of such cell types. This latter situation is more common and considered to be more appropriate for maximum likelihood estimation (i.e., the prior probabilities of each class are considered equal in Bayesian decision making), which is used as the default option.

If users choose to consider the prior probabilities of different cell types, they can replace the strategy for making decision (formular (****) in part Supervised cell type prediction by SciBet, section METHODS) with the following strategy of Bayes Decision Rule $\hat{j} = argmax_j(P(y|j) * P(j))$= $argmax_j\left(\prod_i\left(p_{ij}{}^{y_i}\right) * P(j)\right)$, to make decisions. And in most cases, users can estimate the prior probability according to the proportion of cell types in the training set.

SUPPLEMENTARY REFERENCES

1 Tabula Muris, C. et al. Single-cell transcriptomics of 20 mouse organs creates a Tabula Muris. Nature 562, 367-372, doi:10.1038/s41586-018-0590-4 (2018).

2 Muraro, M. J. et al. A Single-Cell Transcriptome Atlas of the Human Pancreas. Cell Syst. 3, 385-394.e3 (2016).

3 Baron, M. et al. A Single-Cell Transcriptomic Map of the Human and Mouse Pancreas Reveals Inter- and Intra-cell Population Structure. Cell Syst. 3, 346-360.e4 (2016).

4 Trapnell, C. et al. The dynamics and regulators of cell fate decisions are revealed by pseudotemporal ordering of single cells. Nat. Biotechnol. 32, 381 (2014).

5 Macosko, E. Z. et al. Highly Parallel Genome-wide Expression Profiling of Individual Cells Using Nanoliter Droplets. Cell 161, 1202–1214 (2015).

6 Leng, N. et al. Oscope identifies oscillatory genes in unsynchronized single-cell RNA-seq experiments. Nat. Methods 12, 947 (2015).

7 Hou, Y. et al. Single-cell triple omics sequencing reveals genetic, epigenetic, and transcriptomic heterogeneity in hepatocellular carcinomas. Cell Res. 26, 304 (2016).

8 Padovan-Merhar, O. et al. Single Mammalian Cells Compensate for Differences in Cellular Volume and DNA Copy Number through Independent Global Transcriptional Mechanisms. Mol. Cell 58, 339–352 (2015).

9 Darmanis, S. et al. A survey of human brain transcriptome diversity at the single cell level. Proc. Natl. Acad. Sci. 112, 7285 (2015).

10 Björklund, Å. K. et al. The heterogeneity of human CD127+ innate lymphoid cells revealed by single-cell RNA sequencing. Nat. Immunol. 17, 451 (2016).

11 Camp, J. G. et al. Human cerebral organoids recapitulate gene expression programs of fetal neocortex development. Proc. Natl. Acad. Sci. 112, 15672 (2015).

12 Velten, L. et al. Human haematopoietic stem cell lineage commitment is a continuous process. Nat. Cell Biol. 19, 271 (2017).

13 Chu, L.-F. et al. Single-cell RNA-seq reveals novel regulators of human embryonic stem cell

differentiation to definitive endoderm. Genome Biol. 17, 173 (2016).

14 Tung, P.-Y. et al. Batch effects and the effective design of single-cell gene expression studies. Sci. Rep. 7, 39921 (2017).

15 Zeng, W. et al. Single-nucleus RNA-seq of differentiating human myoblasts reveals the extent of fate heterogeneity. Nucleic Acids Res. 44, e158–e158 (2016).

16 Camp, J. G. et al. Multilineage communication regulates human liver bud development from pluripotency. Nature 546, 533 (2017).

17 Xin, Y. et al. RNA Sequencing of Single Human Islet Cells Reveals Type 2 Diabetes Genes. Cell Metab. 24, 608–615 (2016).

18 Wang, Y. J. et al. Single-Cell Transcriptomics of the Human Endocrine Pancreas. Diabetes 65, 3028 (2016).

19 Mora-Bermúdez, F. et al. Differences and similarities between human and chimpanzee neural progenitors during cerebral cortex development. eLife 5, e18683 (2016).

20 Xu, Y. et al. Single-cell RNA sequencing identifies diverse roles of epithelial cells in idiopathic pulmonary fibrosis. JCI Insight 1, (2017).

21 Yao, Z. et al. A Single-Cell Roadmap of Lineage Bifurcation in Human ESC Models of Embryonic Brain Development. Cell Stem Cell 20, 120–134 (2017).

22 Sahakyan, A. et al. Human Naive Pluripotent Stem Cells Model X Chromosome Dampening and X Inactivation. Cell Stem Cell 20, 87–101 (2017).

23 Wills, Q. F. et al. The nature and nurture of cell heterogeneity: accounting for macrophage gene-environment interactions with single-cell RNA-Seq. BMC Genomics 18, 53–53 (2017).

24 Breton, G. et al. Human dendritic cells (DCs) are derived from distinct circulating precursors that are precommitted to become CD1c+ or CD141+ DCs. J. Exp. Med. 213, 2861–2870 (2016).

25 Arguel, M.-J. et al. A cost effective 5´ selective single cell transcriptome profiling approach with improved UMI design. Nucleic Acids Res. 45, e48–e48 (2017).

26 Enge, M. et al. Single-Cell Analysis of Human Pancreas Reveals Transcriptional Signatures of Aging and Somatic Mutation Patterns. Cell 171, 321-330 e314, doi:10.1016/j.cell.2017.09.004 (2017).

27 Close, J. L. et al. Single-Cell Profiling of an In Vitro Model of Human Interneuron Development Reveals Temporal Dynamics of Cell Type Production and Maturation. Neuron 93, 1035-1048.e5

(2017).

28 Zheng, G. X. Y. et al. Massively parallel digital transcriptional profiling of single cells. Nat. Commun. 8, 14049 (2017).

29 Zhong, S. et al. A single-cell RNA-seq survey of the developmental landscape of the human prefrontal cortex. Nature 555, 524–528 (2018).

30 Li, L. et al. Single-Cell RNA-Seq Analysis Maps Development of Human Germline Cells and Gonadal Niche Interactions. Cell Stem Cell 20, 858-873.e4 (2017).

31 Azizi, E. et al. Single-Cell Map of Diverse Immune Phenotypes in the Breast Tumor Microenvironment. Cell 174, 1293-1308.e36 (2018).

32 Guo, X. et al. Global characterization of T cells in non-small-cell lung cancer by single-cell sequencing. Nat. Med. 24, 978–985 (2018).

33 Zhang, L. et al. Lineage tracking reveals dynamic relationships of T cells in colorectal cancer. Nature 564, 268–272 (2018).

34 Yan, L. et al. Single-cell RNA-Seq profiling of human preimplantation embryos and embryonic stem cells. Nat. Struct. Amp Mol. Biol. 20, 1131 (2013).

35 Chung, W. et al. Single-cell RNA-seq enables comprehensive tumour and immune cell profiling in primary breast cancer. Nat Commun 8, 15081, doi:10.1038/ncomms15081 (2017).

36 Lizotte, P. H. et al. Multiparametric profiling of non–small-cell lung cancers reveals distinct immunophenotypes. JCI Insight 1, (2016).

37 Tirosh, I. et al. Dissecting the multicellular ecosystem of metastatic melanoma by single-cell RNA-seq. Science 352, 189 (2016).

38 Ohta, R. et al. Laminin-guided highly efficient endothelial commitment from human pluripotent stem cells. Sci. Rep. 6, 35680 (2016).

39 Segerstolpe, Å. et al. Single-Cell Transcriptome Profiling of Human Pancreatic Islets in Health and Type 2 Diabetes. Cell Metab. 24, 593–607 (2016).

40 Gao, S. et al. Tracing the temporal-spatial transcriptome landscapes of the human fetal digestive tract using single-cell RNA-sequencing. Nat. Cell Biol. 20, 721–734 (2018).

41 See, P., Lum, J., Chen, J. & Ginhoux, F. A Single-Cell Sequencing Guide for Immunologists. Front. Immunol. 9, 2425 (2018).

42 La Manno, G. et al. Molecular Diversity of Midbrain Development in Mouse, Human, and Stem

Cells. Cell 167, 566-580.e19 (2016).

43 Zheng, C. et al. Landscape of Infiltrating T Cells in Liver Cancer Revealed by Single-Cell Sequencing. Cell 169, 1342-1356 e1316, doi:10.1016/j.cell.2017.05.035 (2017).

44 Pedregosa, F. *et al.* Scikit-learn: Machine Learning in Python. *J Mach Learn Res* **12**, 2825-2830 (2011).

45 Zappia, L., Phipson, B. & Oshlack, A. Splatter: simulation of single-cell RNA sequencing data. Genome Biol 18, 174, doi:10.1186/s13059-017-1305-0 (2017).

46 Huang, M. et al. SAVER: gene expression recovery for single-cell RNA sequencing. Nat Methods 15, 539-542, doi:10.1038/s41592-018-0033-z (2018).

47 Awad, A. The Shannon entropy of generalized gamma and of related distribution. (1991).

48 Lun, A. T., Bach, K. & Marioni, J. C. Pooling across cells to normalize single-cell RNA sequencing data with many zero counts. Genome Biol 17, 75, doi:10.1186/s13059-016-0947-7 (2016).
